# Efficacy and safety of bromfenac 0.09% and sodium hyaluronate 0.4% combination therapy, versus placebo in patients with pterygium I–III for clinical signs on ocular inflammation

**DOI:** 10.2147/OPTH.S203648

**Published:** 2019-05-02

**Authors:** Eduardo Chávez-Mondragón, Claudia Palacio, Abraham Soto-Gómez, Miguel Villanueva-Nájera, Guillermo De Wit-Carter, Ruben Suárez-Velasco, Leopoldo Baiza-Duran, Oscar Olvera-Montaño, Patricia Muñoz-Villegas

**Affiliations:** 1 Fundación de Asistencia Privada Conde de Valenciana, IAP, CDMX, Mexico; 2 Fundación Hospital Nuestra Señora de la Luz, IAP, CDMX, Mexico; 3 Catarata y Glaucoma de Occidente, SA de CV, Guadalajara, Jalisco, Mexico; 4 Private Office, CDMX, Mexico; 5 Asociación para Evitar la Ceguera en Mexico IAP, CDMX, Mexico; 6 Novam y Vita, Guadalajara, Jalisco, Mexico; 7 Clinical Research Department, Laboratorios Sophia, SA de CV, Zapopan, Jalisco, Mexico

**Keywords:** nonsteroidal anti-inflammatory drug, Ocular Surface Disease Index, conjunctival hyperemia, ocular lubricant

## Abstract

**Purpose:** This study evaluated the clinical efficacy and safety of bromfenac 0.09%, sodium hyaluronate 0.4% (SH) combination therapy, versus placebo and SH in a clinical model of pterygium I–III.

**Methods:** A total of 166 eyes (99 patients) with pterygium grade I–III were randomized to bromfenac 0.09% ophthalmic solution+SH 0.4% or placebo+SH 0.4%. This was a Phase IV, prospective, parallel, double-masked, multicenter clinical trial. One drop of bromfenac or placebo was instilled two times a day (BID) for 20 days, both groups accompanied treatments with one drop of SH three times a day (TID). The primary efficacy endpoints were the conjunctival hyperemia and the Ocular Surface Disease Index (OSDI) score. Other results measured included burning, foreign body sensation, and photophobia. The safety was assessed by the tear break-up time (TBUT), visual acuity (VA), IOP, lissamine green, fluorescein stains, and the incidence of adverse events (AEs).

**Results:** Compared with baseline, there was a significant reduction in the conjunctival hyperemia (*p*=0.0001) and OSDI score in both groups (*p*=0.0001). There was a significant improvement in ocular symptomatology for both, placebo/SH and bromfenac/SH groups (*p*=0.0001), the decrement in the ocular burning was 41.1% vs 24.6%, the foreign body sensation was 31.5% vs 36.2% and, for photophobia was 23.3% vs 30.5%, respectively. A statistically significant difference was observed in TBUT for bromfenac/SH (*p*=0.045), at day 20. There were no significant alterations in IOP (*p*=0.068) or VA (*p*=0.632). Similar improvements were observed in the fluorescein and green lissamine staining. Finally, the incidence of AE was similar between groups.

**Conclusion:** The treatment with bromfenac 0.09% ophthalmic solution and SH 0.4% combination therapy for 3 weeks reduced clinical signs, in patients with pterygium I–III. The results suggest that bromfenac 0.09% can improve the symptomatology, reduce the presentation of clinical signs associated with superficial ocular inflammation.

## Introduction

Bromfenac is a topical, NSAID. Its chemical structure lengthens the absorption into the ocular tissues and enhances the duration of anti-inflammatory activity.^1^ It is the first and only topical ophthalmic NSAID with a once-daily dosing regimen approved by the Food and Drugs Administration for the treatment of post-operative pain and inflammation following cataract surgery.^2^

The manipulation of ocular structures like surgery, infections, ocular trauma, among others, can produce an inflammatory process. After lysis, multiple chemical mediators induce the expression of cyclooxygenase (COX), an important group of enzymes active in the inflammatory process. Prostaglandins (PGs) are products of COX-2 and have several effects in the tissue. COX-2, which is inducible, is upregulated in states of inflammation, and converts the arachidonic acid into several PGs.^1^–^3^ NSAIDs block the PGs response through inhibiting COX enzymes.^4^–^6^ Furthermore, NSAIDs can decrease pain and photophobia after refractive surgery and alleviate itching associated with allergic conjunctivitis.^5^

Pterygium is a type of benign uncontrolled growth of the conjunctive tissue that lays over the cornea. Pterygium is an inflammatory active, invasive, and chronic disease. However, its pathogenesis remains incompletely understood. Clinically, the condition involves invasive centripetal growth with associated inflammation and neovascularization.^3^ Inflammation induces angiogenic pathways, resulting in neovascularization contributing to pterygia development and growth. COX-2 is expressed in both primary pterygia and recurrent pterygia.^4^

Many patients show foreign body sensation, tearing, chronic ocular discomfort, itching, and redness, and are commonly treated with lubricants, vasoconstrictors, or corticosteroids.^7^Redness can be taken as sign of inflammation which may suggest the progression and severity of a specific disease like pterygium.^8^,^9^ This fibrovascular tissue is characterized by its wedge-shaped and vascular growth that originate in the eye corneal limbus.^9^,^10^

The effectiveness and safety of bromfenac 0.09% ophthalmic solution (Zebesten®, Laboratorios Sophia, SA de CV, Zapopan, Jalisco, Mexico) in the treatment of ocular inflammatory signs and in the reduction of cystoid macular edema after phacoemulsification has been shown previously in Mexican population.^6^

Finally, sodium hyaluronate (SH) has been used as a lubricant. Lubricants have a rheological profile, they are viscous under static conditions on the eye, while this viscosity decreases during blinking; this behavior can be reproduced by hyaluronic acid (sodium salt) eye drops.^11^ Many studies have demonstrated the safety and efficacy of SH as eye drops.^12^–^15^ It was also proposed that SH might have a direct role in the control of ocular surface inflammation. The use of hypotonic SH eye drops has been shown increased tear osmolarity that could play a part in the pathogenesis of the ocular surface damage.^16^ Several clinical symptoms related to pterygium are commonly treated with lubricants.

The purpose of this study was to evaluate the clinical efficacy and safety of bromfenac 0.09% and SH 0.4% ophthalmic solutions in a combination therapy, versus placebo and SH 0.4%, in the presence of clinical signs and ocular inflammation on a clinical model of pterygium I–III.

## Material and methods

### Study design

This was a Phase IV, prospective, parallel, double-masked, multicenter clinical trial (registered at ClinicalTrials.gov as NCT03521791). It was conducted in six centers in Mexico. An ethics committee in each center reviewed and approved the study (see Ethics approval section). The research was conducted in compliance with the Declaration of Helsinki and in accordance with Good Clinical Practices Standards. All patients that participated in this study provided written and signed informed consent. Patients were recruited between January and August 2018.

### Participants

Inclusion criteria included either men or women (aged >18 years) with a diagnosis of pterygium I–III (temporal and/or nasal) according to the Johnston Classification (Table 1).^17^ In the cases where subjects presented bilateral pterygium, both eyes were eligible. Double-headed pterygium cases were eligible too. Exclusion criteria included use of topical ocular drops and systemic medication that may affect the study’s results (eg, NSAID, antihistaminic, artificial tears with BAK, etc.), patients with autoimmune disease, corneal dystrophy, diagnosis of pterygium IV, corneal dellen, ocular surface inflammatory, proliferative or neoplastic diseases, any type of corneal ulcers, history of penetrating keratoplasty, ocular surgery within 3 months before baseline, pregnant or breastfeeding patients and patients at risk of pregnancy without birth control treatment.

**Table 1:** Pterygium classification

Stage 0	Pingeuculum, posterior to the Limbus.
Stage I	Tissue involvement to the Limbus.
Stage II	Tissue just on to the Limbus.
Stage III	Tissue between the Limbus and Pupillary Margin.
Stage IV	Tissue central to the Pupillary Margin.

### Treatment and evaluations

A total of 166 eyes (99 patients) were randomized 1:1 to bromfenac 0.09% ophthalmic solution (PRO-155)+SH 0.4% (Lagricel® Ofteno, Laboratorios Sophia, SA de CV, Zapopan, Jalisco, Mexico) (n=47 subjects) or placebo+SH 0.4% (n=52 subjects), using a random numbers software. Patients instilled one drop of study drug in the inferior conjunctival sac (PRO-155 or placebo) BID and one drop TID of SH 0.4% for 20 days. Treatment assignment was masked throughout the study for all the researchers, patients, and other sponsoring team members. Follow-up visits were on days 7, 15, and 21 after randomization. A safety call was carried out 2 weeks after the final visit (36th day). The study drug was discontinued if either the principal investigator or patient judged that it was not in the latter’s best interest to continue.

At each follow-up visit, tear break-up time (TBUT), Ocular Surface Disease Index (OSDI), slit-lamp examination, visual acuity, and fluorescein and green lissamine dyes were examined.

Subjective symptoms were graded on a numerical scale of 0–4 using the OSDI questionnaire.^18^ The sum of the scores was used in the analysis. The TBUT was recorded as the number of seconds that elapsed between the last blink and the appearance of the first dry spot in the tear film. The ocular surface was evaluated with a slit-lamp aided by fluorescein staining, and for conjunctival staining, green lissamine was instilled in the eye. Surface dye staining was classified in a scale from 0 to V in accordance with the percentage of the affected area (Oxford scale). The visual acuity was determined with a Snellen chart and expressed in LogMAR values. The IOP was measured using a calibrated Goldmann applanation tonometer during the baseline and final visits.

The primary efficacy endpoints were the conjunctival hyperemia and the OSDI score. The tolerability was assessed by the burning, foreign body sensation, and photophobia. The safety was measured by the TBUT, visual acuity, IOP, lissamine green and fluorescein ocular surface stains, and the incidence of adverse events (AEs).

### Statistical analysis

Statistical analysis was carried out using SPSS 19.0 software (SPSS Inc., Chicago, IL, USA). Efficacy evaluations were made only in per-protocol population (PP), established as a randomized patient with no major deviation from the protocol after performing a bivariate analysis. Sample size calculation was performed to test the improvement of conjunctival hyperemia after pterygium surgery of 2.9 grades of severity.^19^ With an alpha of 0.05, a power of 80%, and standard deviation of 0.4, a sample size of 69 cases (eyes) was found to be necessary. It was therefore planned to include 83 eyes per group in this study, allowing as much as 20% of cases to be excluded from the PP population as a result of major protocol deviations. Comparisons of treatment group categorical variables were done using the Mann–Whitney U test or Fisher’s exact test. The continuous variables were analyzed using Student's *t*-test and Chi-square test. The ordinal variables were analyzed using 2×2 contingency tables (McNemar’s test). The Wilcoxon signed rank test was used to compare within-group categorical variable changes from the baseline value. A two-side test with p≤0.05 was considered to indicate statistical significance.

## Results

### Characteristics of the participants

Ninety-nine patients were enrolled. Of the 99 patients, 42 (80.8%) patients in group 1 (placebo/SH), and 41 (87.2%) patients in group 2 (PRO-155/SH) completed the entire protocol without deviations. Between 19.2% and 12.8% of the patients discontinued their participation secondary to: presentation of AE (1 patient, group 1), patient’s decision unrelated to AE (2 patients, group 1), lost of follow-up (4 patients, group 2), and protocol deviations (7 and 2 patients, respectively). Demographic and baseline characteristic were similar between the two treatment groups without significant differences, see Table 2. Mean age±SD was 55.5±13.4 years (range 27–87); 72.3% of patients were female. Clinical signs and symptoms were similar between groups.

**Table 2:** Demographic and baseline characteristics (n=83 completed patients)

	Placebo/SH	PRO-155/SH	*p*
Female, n (%)	30 (71.4)	30 (73.2)	1.000^a^
Male, n (%)	12 (28.6)	11 (26.8)	1.000^a^
Age (years)±SD	57.2±14.4	53.8±12.3	0.348^b^
IOP (mmHg)±SD	13.03±2.0	13.25±2.5	0.615^b^
TBUT (seconds)±SD	7.04±2.3	6.93±2.9	0.578^b^
OSD±SD	30.3±23.7	22.5±17.6	0.167^b^
Mean visual acuity (LogMAR)±SD	0.15±0.2	0.16±0.2	0.535^b^

**Notes:** ^a^Fisher’s exact test, ^b^Mann–Whitney *U*. **Abbreviations:** SH, sodium hyaluronate; PRO-155, bromfenac; TBUT, Tear break-up time; OSDI, ocular surface disease index.

### Efficacy

#### Conjunctival hyperemia

**Figure 1:**
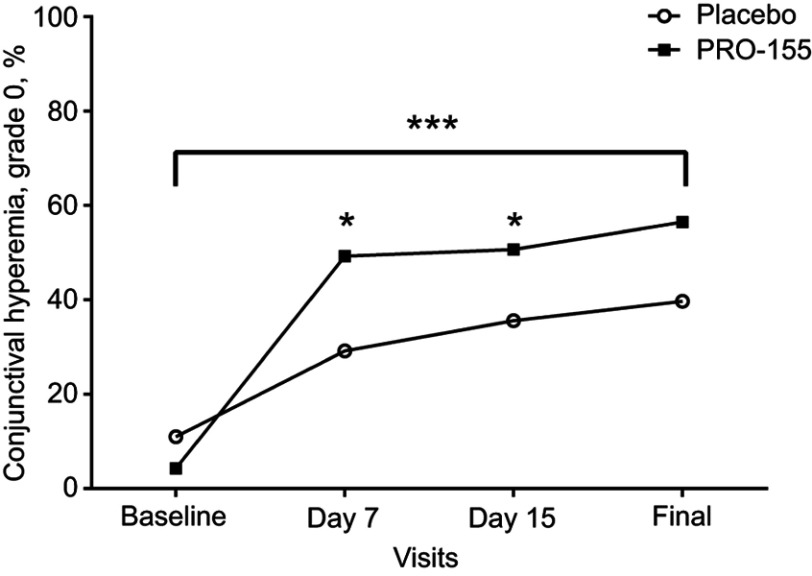
Conjunctival hyperemia. Frequency of conjunctival hyperemia, grade 0 (normal). At each experimental visit for placebo/SH (black circle) and PRO-155/SH (white square). ***p=0.0001 for final visit>baseline (McNemar’s test), *p<0.05 for PRO-155/SH>placebo/SH at days 7 and 15 (*X*^2^ test).

In the PP population, after 3 weeks of treatment, compared with the baseline there was a significant reduction in the conjunctival hyperemia in both groups (*p*=0.0001). The patients treated with placebo/SH resulted in a reduction of the severity on Efron scale a 29% (slight severity). This improvement was higher in the PRO-155/SH group, when a significant reduction on the severity was found in the final visit over the 52% of the patients. On the day 7 (visit 1), and on day 15 (visit 2) the improvement on the PRO-155/SH was statistically significant when compared to Placebo/SH (*p*=0.049 and *p*=0.021, respectively), see Figure 1.

#### Ocular Surface Disease Index (OSDI)

Baseline OSDI was similar between groups (placebo/SH: 30.31±23.7 and PRO-155/SH: 22.51±17.6, *p*=0.167). There was a statistically significant reduction in the OSDI score in both groups during their final visit (12.35±16.8 vs 6.25±8.2, respectively) compared with the baseline (*p*=0.0001). No difference was observed between the two groups (*p*=0.086), see Table 4.

**Table 3:** Ocular symptomatology (presence)

Symptom	Placebo/SH (n=73)		PRO-155/SH (n=69)	
Baseline	Final	Baseline	Final
Burning, %	56.2	15.1	36.2	11.6
Foreign body sensation, %	56.2	24.7	50.7	14.5
Photophobia, %	39.7	16.4	34.8	4.3

**Notes:** Baseline vs Final visit, McNemar’s test *p*=0.0001. **Abbreviations:** SH, sodium hyaluronate; PRO-155, bromfenac.

**Table 4:** Clinical signs and safety

	Placebo/SH	PRO-155/SH	*p*
Lissamine green staining (Grade 0–I), %	89.0	89.9	0.117^a^
Fluorescein staining (Grade 0–I), %	87.7	81.2	0.355^b^
*TBUT, seconds	7.63±2.3	8.29±2.2	0.045^c^
*OSDI score	12.35±16.8	6.25±8.2	0.086^c^
*IOP, mmHg	13.25±2.5	12.45±2.1	0.068^c^
*Mean visual acuity, LogMAR	0.06±0.2	0.04±0.1	0.319^c^
AEs, %	11.5	6.4	0.492^a^

**Notes:** *Data is presented in mean±standard deviation. ^a^Chi^2^ test, ^b^Fisher’s exact test, ^c^Mann–Whitney *U*. **Abbreviations:** SH, sodium hyaluronate; PRO-155, bromfenac; AEs, adverse events; TBUT, tear break-up time; OSDI, ocular surface disease index.

### Tolerability

#### Ocular symptomatology

Burning, foreign body sensation, and photophobia were considered the parameters of ocular symptomatology. All subjects showed a significant improvement of ocular symptomatology over the duration of the study. As shown in Table 3, there was a significant decrease of burning, foreign body sensation, and photophobia from the baseline to final visit (*p*=0.0001) in both groups. At the end of the study, the improvement in ocular burning for the placebo/SH group was 41.1%, for foreign body sensation it was 31.5% and, for photophobia it was 23.3%. The improvement in the ocular symptomatology for the PRO-155/SH group was 24.6%, 36.2%, and 30.5%, respectively. The improvement in photophobia was higher in the PRO-155/SH group (*p*=0.027).

### Safety

#### Tear break-up time (TBUT)

**Figure 2:**
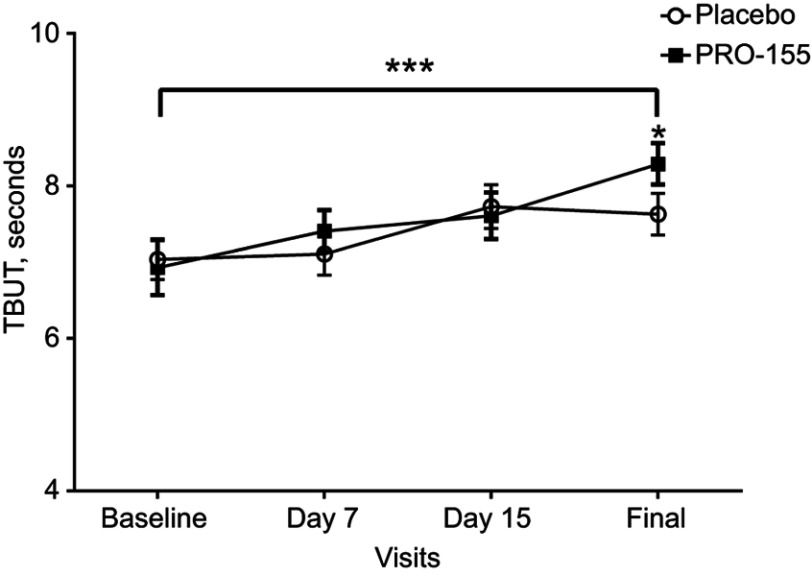
Tear break-up time. Tear break-up time±S.E.M. At each experimental visit for placebo/SH (black circle) and PRO-155/SH (white square). ***p=0.0001 for final visit>baseline (Wilcoxon test), *p=0.045 for PRO-155/SH>placebo/SH at final visit (Mann–Whitney *U* test).

After the intervention, the TBUT increased in both groups compared with baseline (*p*=0.0001), with a significant difference between treatments in the final visit (7.63±2.3 vs 8.29±2.2), when PRO-155/SH was higher (*p*=0.045), see Figure 2.

#### Visual acuity (LogMAR)

The visual acuity did not decrease from baseline (0.15±0.2 vs 0.16±0.2) to final visit (0.14±0.2 vs 0.13±0.2) in placebo/SH and PRO-155/SH, respectively (*p*=0.101), see Table 4.

#### IOP

After the intervention period, the IOP did not increase from baseline (13.03±2.1 vs 13.25±2.5) to final visit (13.08±1.8 vs 12.45±2.1) for placebo/SH and PRO-155/SH groups, respectively (*p*=0.068), see Table 4.

#### Fluorescein and green lissamine staining

There was a statistically significant improvement for the fluorescein staining score in both groups (*p*=0.0001). The fluorescein staining score change from baseline for the placebo/SH group was 27.1% at day 7, 37% at day 15, and 31.5% at the final visit. Meanwhile, the improvement from baseline for PRO-155/SH was 17.4%, 26.1%, and 16%, respectively. No difference was observed between the two groups. Similar findings in the green lissamine staining were observed. There was a statistically significant improvement in both groups (*p*=0.0001). The green lissamine staining score for the placebo/SH group was 19% at day 7, 23.3% at day 15, and 19.1% at the final visit. Meanwhile, the improvement for PRO-155/SH was 10.1%, 20.3%, and 20.3%, respectively. No significant between-group differences were observed, see Table 4.

#### Adverse events (AEs)

Data on safety was analyzed for the to-be-treated population (ITT). A total of 20 AE were reported by 9.1% (9/99) of the randomized patients during the protocol. There were no significant differences for the incidence of AE between treatments (*X*^2^_(1)_=0.794, *p*=0.492). A total of 17 AE were reported for placebo/SH (14 ocular-EA and 3 non-ocular), and 3 for PRO-155/SH (1 ocular-EA and 2 non-ocular), there were no significant differences for the ocular or non-ocular AE between treatments (*X*^2^_(1)_=3.268, *p*=0.140. The most commonly reported non-ocular AE was headache (5%), while the most frequent ocular AE were ocular pain (15%) and foreign body sensation (15%). Serious AE did not occur during the study.

## Discussion

Pterygium is an inflammatory, active, invasive, and chronic disease of unclear pathogenesis. Inflammation is the manifestation of vascular and cellular response of the tissue to injury.^1^,^3^ Bromfenac is a topical, NSAID, its chemical structure lengthens the absorption into the ocular tissues and enhances the duration of anti-inflammatory activity.^1^ Previous studies have been conducted to evaluate the safety and efficacy of bromfenac 0.09% for the treatment of signs associated to ocular inflammation and for the reduction of presentation of cystoid macular edema after phacoemulsification.^6^ Several clinical symptoms related to pterygium are commonly treated with lubricants. SH has been used as a lubricant, its use could play an important part in the pathogenesis of the ocular surface damage. SH increase precorneal tear film stability, reduce the tear evaporation rate, and the use of hypotonic hyaluronate should be encouraged for the treatment of diseases of ocular surface.^7^,^16^

In this study, we compared the efficacy and safety of bromfenac 0.09% and SH 0.4% combination therapy, versus placebo in a clinical model of pterygium I–III. We demonstrated that the combination of bromfenac and SH effectively reduces the presence of clinical signs of ocular inflammation. Fibrovascular redness is an important clinical sign, characterized by its wedge-shaped, angular and vascular growth. Also, redness can be taken as a sign of inflammation.^9^,^10^ After 3 weeks of treatment, there was a significant decrease in the conjunctival hyperemia in both groups. As much as 29% of patients in group 1 (placebo/SH) improved the severity of their clinical presentation. The results indicate that SH has a beneficial effect on the treatment of conjunctival hyperemia, in agreement with other studies conducted with SH eye drops.^14^,^16^ Meanwhile, 52% of group 2 (PRO-155/SH) showed a decrease in the severity of their conjunctival hyperemia, patients in group 2 reported a significantly greater improvement in conjunctival hyperemia compared with placebo group at day 7; this result is consistent with previous studies.^6^

The OSDI is a valid and trustworthy instrument for measuring of the symptoms of ocular irritation related to dry eye disease and their impact on vision-related function. It possesses necessary psychometric properties to be used as an end point in clinical trials.^18^ In this study, subjective symptoms were graded using the OSDI questionnaire. Both groups demonstrated a reduction of OSDI scores which reached normal values by the final visit.

Likewise, many patients with pterygium show foreign body sensation, tearing, chronic ocular discomfort, itching, and redness.^7^ In our study, clinical symptoms like burning, foreign body sensation, and photophobia were significantly decreased by the final visit in both groups. The improvement in photophobia was higher in the PRO-155/SH versus placebo/SH at the end of the study, consistently with previous studies with NSAIDs.^5^

TBUT, visual acuity, IOP and fluorescein, and green lissamine staining were performed to evaluate the safety of bromfenac. Mean TBUT was increased in both groups, with significant differences in PRO-155/SH by the end of the study. For visual acuity and IOP, there were no differences between treatments.

Topical ophthalmic NSAIDs are commonly used as the treatment of post-operative inflammation in several surgical procedures. Local irritation signs and symptoms include conjunctival hyperemia, burning, stinging, and corneal anesthesia. A more serious complication involves corneal ulceration and full-thickness corneal melts.^20^ In our study, regardless of literature reports of AE due to the use of NSAIDs, no differences were shown between treatments for lissamine green and fluorescein staining. Ocular staining scores were lower (Oxford scale) in both groups compared with their baseline, similarly to other authors’ findings on regards to the use of bromfenac.^6^ Similarly, the presence of AEs in both groups showed no statistically significant differences. Only one patient in placebo/SH group discontinued the study due to a non-serious AE. Non-serious AEs occurred during the study.

Many studies have demonstrated the safety and efficacy of SH as a lubricant.^12^–^15^ SH might have a direct role in the control of ocular surface inflammation in patients with dry eye. Meanwhile, bromfenac blocks the PGs response through inhibiting COX enzymes.^4^–^6^ In the present study, the combination bromfenac 0.09% and SH 0.4% was effective in reducing the presence of clinical signs and ocular inflammation on a clinical model of pterygium I–III.

Further studies are necessary to understand the effect of SH in the treatment of clinical signs and ocular inflammation, and trials with a prolonged follow-up are needed to conclusively determine the efficacy of this combination therapy.

## Conclusion

The treatment with the combination of bromfenac 0.09% and SH 0.4% for 3 weeks reduces clinical signs (conjunctival hyperemia, photophobia, OSDI, burning, and foreign body sensation) in patients with pterygium I–III. Furthermore, 0.09% bromfenac/SH 0.4% are more effective than SH 0.4% eye drops alone in improving conjunctival hyperemia, TBUT, and photophobia in pterygium I–III. These results suggest that bromfenac 0.09% can improve the presentation of clinical signs on ocular inflammation.

## Abbreviation list

OSDI, Ocular Surface Disease Index; PGs, prostaglandins; PRO-155, bromfenac 0.09%; SH, sodium hyaluronate; TBUT, tear break-up time.

## Data sharing

The database generated during and/or analyzed during the current study are available from the corresponding author on reasonable request.
